# Supplementary material for: A Novel Gelatin-Based Sustained-Release Molluscicide for Control of the Invasive Agricultural Pest and Disease Vector Pomacea canaliculata
Source: Molecules. 2022 Jul 2;27(13):4268. doi: 10.3390/molecules27134268 (PMC9268488; doi:10.3390/molecules27134268)
Supplement: Supplementary file 1 [file molecules-27-04268-s001.zip › molecules-1735208-supplementary.pdf]

Table S1. Average mortality rates of gelatin including molluscacide on Pomacea canaliculata (%).

Concentration	12h	Mean±SD	24h	Mean±SD	48h	Mean±SD	72h	Mean±SD	96h	Mean±SD
1	53.33	5.77	76.67	5.77	100	0	100	0	100	0
0.5	26.67	11.55	53.33	5.77	96.67	5.77	100	0	100	0
0.1	3.33	5.77	20	10	50	10	83.33	5.77	100	0
0.05	0	0	10	10	30	10	70	10	100	0
0	0	0	0	0	0	0	0	0	3.33	5.77

Table S2. Regression and correlation analysis on toxicity of niclosamide gelatin sustained-release agent to *Pomacea Canaliculata*.

Time	Regression equation	R^2	LC_{50} (mg/L)	95% Confidence interval	R	P value
12h	y = 2.340x-2.123	0.955	0.907	0.685-1.427	0.975	0.005
24 h	y = 2.263x-1.333	0.945	0.589	0.404-0.899	1	<0.001
48 h	y = 6.587x-1.029	0.988	0.156	0.083-0.338	1	<0.001
72 h	y = 18.870x-0.948	1	0.05	0.009-0.087	0.975	0.005
96 h	y = 26.185x-0.651	<0.001	0.025	-0.015~0.051	0.707	0.182

PS: LC50 is 50% lethal concentration.Y is the probability unit of death and x is the concentration of niclosamide gelatin sustained release agent.

Table S3. Average mortality rates of zebrafish exposed to Niclosamide under different temperatures (%)

**Concentration of Niclosamide (mg/L)）**	**2h**	**4h**	**6h**	**8h**	**12h**	**24h**	**48h**	**72h**	**96h**	**120h**	**144h**	**168h**	**192h**
1 (gelatin)	0	0	0	0	0	0	0	6	20	23.3	23.3	23.3	23.3
0.5 (gelatin)	0	0	0	0	0	0	0	3	10	13.3	13.3	13.3	13.3
0.1 (gelatin)	0	0	0	0	0	0	0	0	0	0	0	0	0
0.05 (gelatin)）	0	0	0	0	0	0	0	0	0	0	0	0	0
0 (gelatin)	0	0	0	0	0	0	0	0	0	0	0	0	0
1 (solution)	100	100	100	100	100	100	100	100	100	100	100	100	100
0.5 (solution)	100	100	100	100	100	100	100	100	100	100	100	100	100
0.1 (solution)	0	0	0	20	50	70	90	100	100	100	100	100	100
0.05 (solution)	0	0	0	0	0	0	10	20	50	50	50	50	50

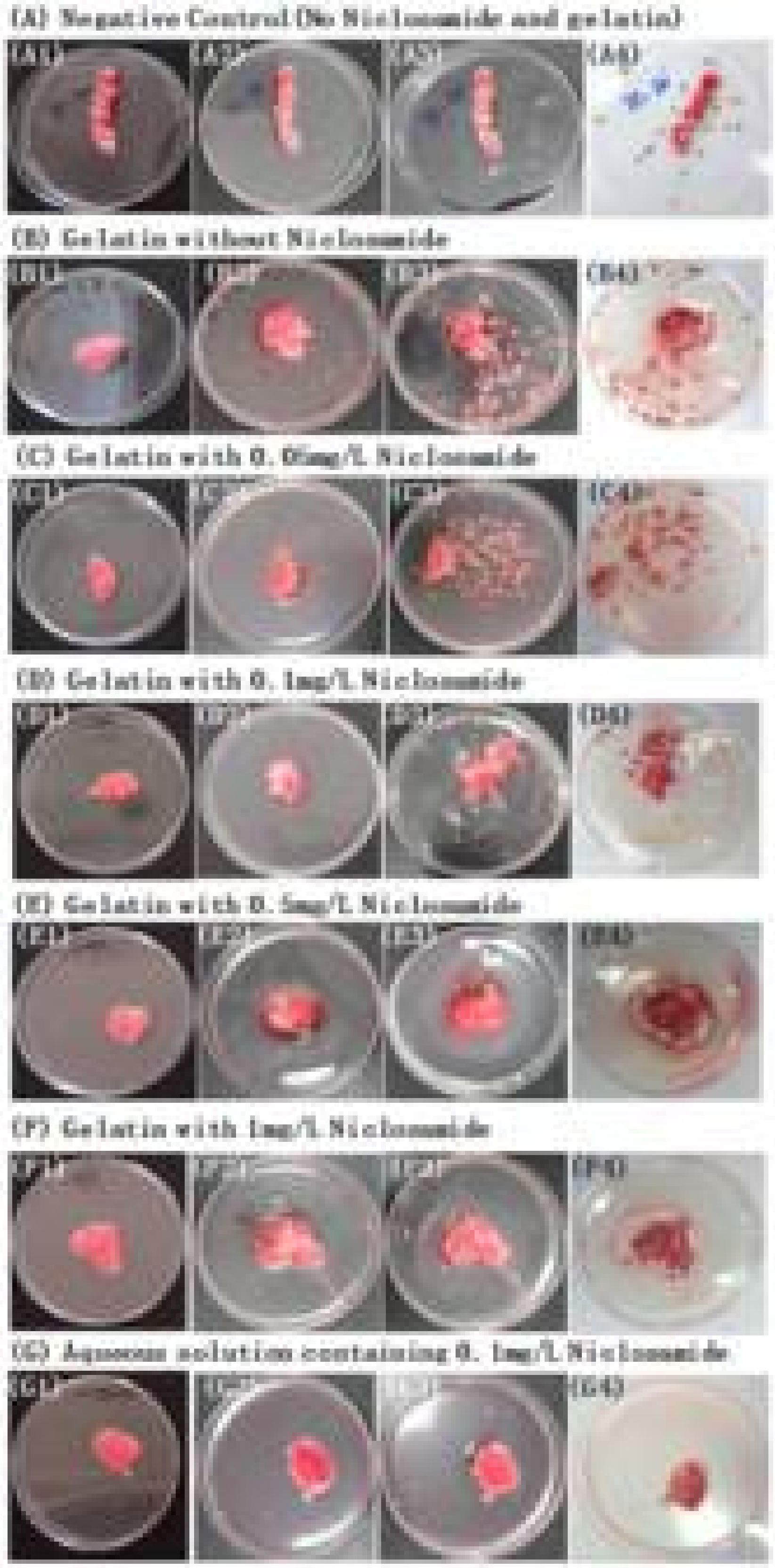

Figure S1. A1, A2, A3, and A4 represent *P. canaliculata* eggs without niclosamide and gelatin treatment at 0, 5, 10, and 15 days. (B) B1, B2, B3, and B4 represent *P. canaliculata* eggs treated with gelatin with no niclosamide for 0, 5, 10, and 15 days. (C) C1, C2, C3, and C4 represent *P. canaliculata* eggs treated using gelatin with 0.05 mg/L niclosamide for 0, 5, 10, and 15 days. (D) D1, D2, D3, and D4 represent *P. canaliculata* eggs treated using gelatin with 0.1 mg/L niclosamide for 0, 5, 10, and 15 days, respectively. (E) E1, E2, E3, and E4 represent *P. canaliculata* eggs treated using gelatin with 0.5 mg/L niclosamide for 0, 5, 10, and 15 days, respectively. (F) F1, F2, F3, and F4 represent *P. canaliculata* eggs treated using gelatin with 1 mg/L niclosamide for 0, 5, 10, and 15 days, respectively. (G) G1,

G2, G3, and G4 represent *P. canaliculata* eggs treated using an aqueous solution with 0.1 mg/L niclosamide for 0, 5, 10, and 15 days, respectively.
